# Utilization of Continuous Cardiac Monitoring on Hospitalist-led Teaching Teams

**DOI:** 10.7759/cureus.3300

**Published:** 2018-09-13

**Authors:** Debbie W Chen, Robert Park, Sarah Young, Divya Chalikonda, Kemarut Laothamatas, Gretchen Diemer

**Affiliations:** 1 Internal Medicine, Thomas Jefferson University Hospitals, Philadelphia, USA; 2 Medicine, Sidney Kimmel Medical College at Thomas Jefferson University, Philadelphia, USA

**Keywords:** telemetry, continuous cardiac monitoring, hospitalist, high value care

## Abstract

Guidelines for continuous cardiac monitoring (CCM) have focused almost exclusively on cardiac diagnoses, thus limiting their application to a general medical population. In this study, a retrospective chart review was performed to identify the reasons that general medical patients, cared for on hospitalist-led inpatient teaching teams between April 2017 and February 2018, were initiated and maintained on CCM, and to determine the incidence of clinically significant arrhythmias in this patient population. The three most common reasons for telemetry initiation were sepsis (24%), arrhythmias (12%), and hypoxia (10%). Most patients remained on telemetry for more than 48 hours (62%) and a significant number of patients were on telemetry until they were discharged from the hospital (39%). Of the cumulative total of more than 20,573 hours of CCM provided to this patient population, 37% of patients demonstrated only normal sinus rhythm and 3% had a clinically significant arrhythmia that affected management.

## Introduction

Telemetry was introduced in hospitals during the 1960s to provide continuous cardiac monitoring (CCM) in cardiac intensive care units (ICUs). Over the last five decades, the use of telemetry has expanded beyond the ICU setting to non-critical care settings [1]. Concurrent with the broad application of cardiac monitoring, the American Heart Association (AHA) and American College of Cardiology (ACC) have published guidelines for the appropriate use of non-ICU electrocardiographic (ECG) monitoring based on a consensus opinion [1-2]. Despite the existence of these guidelines, numerous studies have shown that a significant number of non-ICU general medical patients on CCM do not meet appropriate indications, with a resultant low incidence of arrhythmias detected [3-5].

The inappropriate use of telemetry monitoring can negatively affect patients, providers, and the hospital organization. Cardiac monitoring is expensive, given the cost of equipment, maintenance, and supplies, including batteries, paper, and monitor leads. It is also labor-intensive; nurses spend an average of about 20 minutes per patient per day on telemetry-related tasks [6]. Telemetry monitoring is sometimes associated with unnecessary testing and intervention, especially if there is a misinterpretation of the waveform artifact. For example, monitor leads can exacerbate underlying delirium in elderly patients [7]. The excessive alarms of cardiac monitoring can be distracting, interfere with patient care, and contribute to alarm fatigue [8]. In addition, the inappropriate allocation of limited telemetry-capable beds creates a bottleneck to the timely disposition of patients from the emergency department. In all, the overutilization of non-indicated cardiac monitoring contributes to a system of waste in hospitals.

The 1991 ACC and 2004 AHA guidelines for telemetry utilization focus almost exclusively on cardiac diagnoses, thus limiting its application to a general medical population. Few studies have examined telemetry utilization among general medical patients. Najafi and Auerbach found that at the University of California San Francisco Medical Center, physicians’ concern for clinical deterioration rather than an explicit concern for arrhythmia detection drove most of the telemetry use for this population [9]. However, no study has specifically examined telemetry utilization among non-ICU general medical patients who were initiated on telemetry after being admitted to the hospital, a decision that is often made by the primary inpatient team. Such studies are essential to better understand practice patterns associated with CCM among hospitalists and can contribute to the development of focused interventions that aim to decrease the overutilization of this limited resource.

The aim of this study was to identify the reasons that non-ICU general medical patients cared for on hospitalist-led inpatient teaching teams were initiated and maintained on CCM and to determine the incidence of clinically significant arrhythmias in this patient population.

## Materials and methods

Study design

Patients who were admitted to a general internal medicine (IM) team under a level of care (LOC) of "General Floor" without telemetry between April 2017, which coincided with a hospital-wide transition to the Epic electronic medical record (Epic Systems Corporation, Wisconsin, US), and February 2018 were identified. A retrospective chart review was performed on those patients who were subsequently initiated on CCM with a change in LOC to "telemetry." The time of order entry for admission/LOC changes/discharge and comprehensive clinical data, including demographic information, medical/surgical/social history, medications, primary diagnoses, electrocardiogram (ECG) results, and length of stay was collected. Indication for the initiation of CCM was determined from comments written in the CCM order and a review of the electronic medical record. All progress notes related to the hospitalization and the discharge summary were reviewed for comments about telemetry results. The study was conducted in accordance with the institutional review board.

Setting

The study was conducted at an urban tertiary care academic medical center where there are seven hospitalist-led IM resident-staffed inpatient teaching teams. These seven teams each consisted of one hospitalist, one senior resident, one or two interns, and up to three medical students. Between April 2017 and February 2018, a total of 21 hospitalists were involved in the care of patients on these seven teams.

The general workflow on these seven hospitalist-led inpatient teaching teams at the time of the study was as follows: between 7 AM and 9 AM, residents pre-rounded on patients. This was followed by patient-centered bedside team rounds, which consisted of the hospitalist attending, residents, staff nurses, a case manager, patients (if able to participate), and families/support persons (if present at the bedside). In the afternoon, the workflow was focused on the completion of tasks related to patient care, discussion about medical management with medical and surgical subspecialty teams if they were consulted, and a reassessment of patients. Between 5 PM and 7 PM, patients were signed out to the overnight team of three residents. Concurrently, between 4 PM and 7 AM, a separate overnight team of four residents admitted patients to these inpatient teaching teams under the supervision of one hospitalist.

Population

Patients who were admitted to a non-telemetry bed on a general IM team between April 2017 and February 2018 then had a subsequent change in the LOC to "telemetry" with the initiation of CCM were identified. The decision to change the LOC to "telemetry" for these patients was made by residents and/or hospitalists on the inpatient teaching teams. Patients who were admitted under the LOC of "telemetry," or admitted to a family medicine/surgical/subspecialist-led inpatient team were not included. Patients admitted to the single hospitalist-led non-teaching team, which was composed of one nurse practitioner and one hospitalist, were also not included because they were medically less complex than patients admitted to the hospitalist-led inpatient teaching teams. At the time of admission, patients on the hospitalist-led non-teaching team were expected to be hemodynamically stable, medically uncomplicated, and discharged within 24-48 hours; patients with hip fractures; and patients with an uncomplicated sickle cell crisis. Exclusion criteria included patients who were transferred to an inpatient team that was not led by a hospitalist; patients who had the order for change in LOC to "telemetry" placed within 30 minutes of the initial "General Floor" admission order by the same health care provider; and patients whose CCM order was discontinued within five minutes of initiation. Patients whose order for a change in LOC to "telemetry" was placed within 30 minutes of the initial "General Floor" admission order were not excluded if the former order was placed by the hospitalist-led admitting team and the latter order by a healthcare professional in the emergency department.

Analysis

Microsoft Excel was used for data analysis. The frequency for each categorical variable was calculated. A univariate analysis was applied to all continuous variables to determine central tendency and dispersion.

## Results

Demographics

One thousand five hundred ninety-four patients were admitted to a hospitalist-led IM resident-staffed inpatient teaching team under a LOC of "general floor" without telemetry between April 2017 and February 2018. Two hundred ninety-six patients were subsequently initiated on CCM during their hospital course with a change in LOC to "telemetry." Forty-two patients were excluded with a resultant study population of 254 patients. The average age was 61.4 + 17 years. Fifty-three percent of patients were male with a Caucasian predominance (n = 117, 46%). Table 1 details the clinical characteristics of the study population.

**Table 1:** Clinical characteristics of the study population

	Overall (n = 254)
Age (years)	61.4 + 17
Male	53% (134)
ETHNICITY	
White or Caucasian	46% (117)
Black or African American	42.5% (108)
Asian, Pacific Islander, or Indian	5% (12)
Hispanic	5% (13)
Unknown	1.5% (4)
MEDICAL HISTORY	
Coronary artery disease	15% (39)
Risk factors for atherosclerosis	
Hypertension	65% (166)
Hypercholesterolemia	42% (107)
Diabetes Mellitus	31% (80)
Atrial fibrillation	11% (27)
Prior personal pacemaker placement	6% (15)
History of a solid tumor cancer	26% (66)
SOCIAL HISTORY	
Current smoker	20% (51)
Alcohol use	33% (83)
Drug use	12% (31)

Telemetry initiation

**Figure 1:**
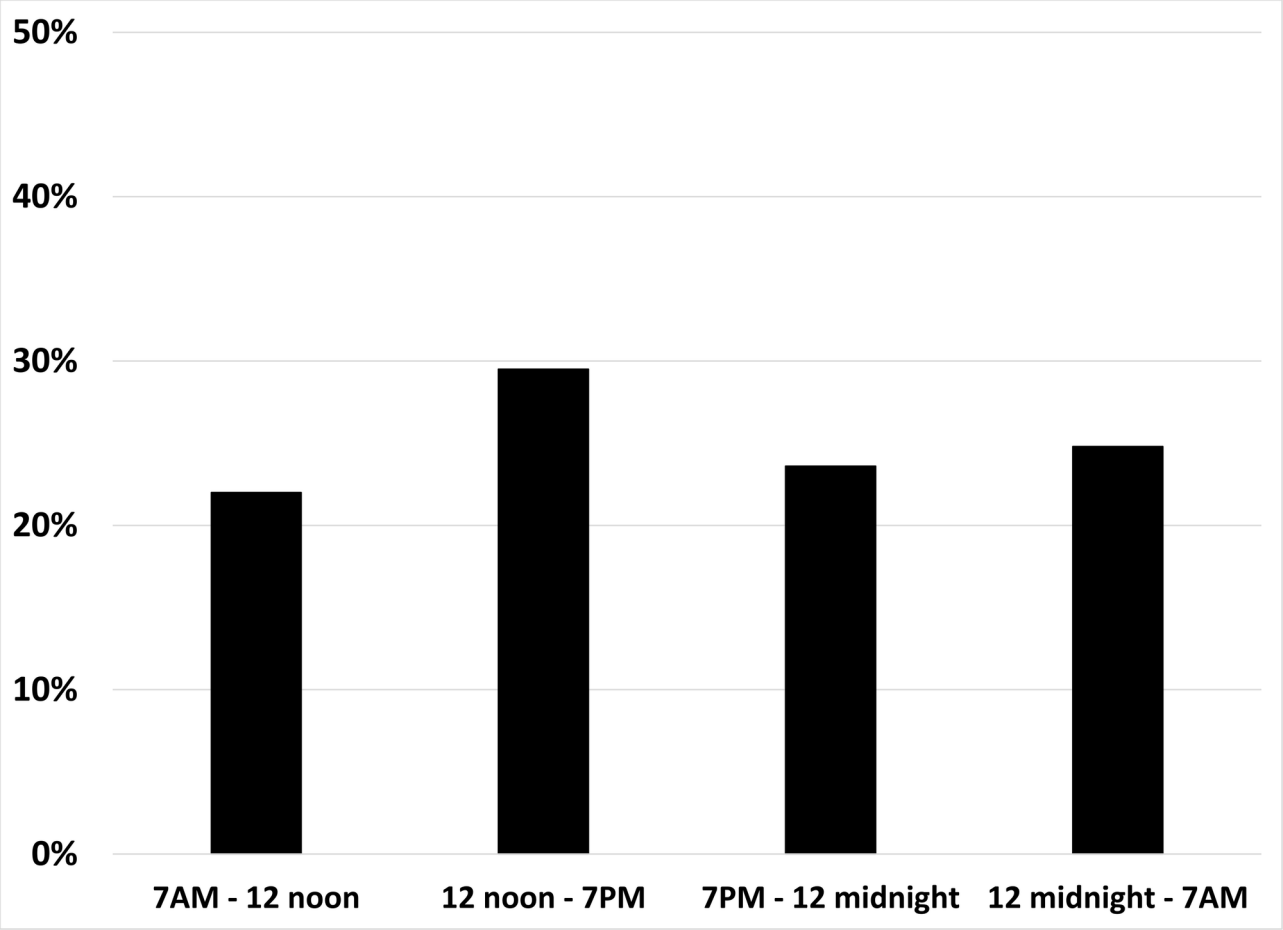
Time of day that continuous cardiac monitoring (CCM) was initiated When patients were cared for by the day team of residents/medical students/ attending hospitalists, 51% of the orders for CCM were placed (22.0% between 7 AM and noon, 29.5% between noon and 7 PM). When most patients were cared for by the overnight residents/supervising hospitalists, 49% of the orders were placed (23.6% between 7 PM and midnight, 24.8% between midnight and 7 AM).

The initiation of patients on telemetry was unaffected by the time of day, team workflow, or coverage provider (day team of residents/medical students/ attending hospitalist or overnight residents/ supervising hospitalist) (Figure 1). The elapsed time between the admission order with a LOC of ‘General Floors’ and the change in LOC to "telemetry" ranged from 0hr:3min to 277hr:21min. The LOC for twenty patients was changed to "telemetry" by the hospitalist-led admitting team within thirty minutes of the initial ‘General Floor’ admission order that was placed by a healthcare professional in the Emergency Department. Seventeen percent of the LOC changes occurred within one hour of the patient being admitted, 49% between one and twelve hours, 13% between twelve and twenty-four hours, and 21% occurred more than twenty-four hours after the patient was admitted. The indication for telemetry varied, but the three most common reasons were sepsis (24%); arrhythmias (12%) such as atrial fibrillation, bradycardia, and tachycardia; and hypoxia (10%) (Table 2). Seventy-three percent of patients (n = 185) were initiated on CCM for non-cardiac reasons.

**Table 2:** Reasons for the initiation of telemetry The reasons for the initiation of continuous cardiac monitoring are listed in descending order of frequency for this patient population. *^a^* Atrial fibrillation (AFib), *^b ^*Rapid ventricular rate (RVR), *^c ^*Atrioventricular (AV), *^d ^*Diabetic Ketoacidosis/Hyperosmolar hyperglycemic state (DKA/HHS), *^e ^*Gastrointestinal (GI). *^f^*Indications included in the "Other" category were: post-implantable cardioverter defibrillator; pericardial effusion; concern for endocarditis; post-operative setting (rib resection, spinal surgery, arteriogram, nephrectomy); concern for drug reaction with eosinophilia and systemic symptoms; stroke concern; biliary obstruction; fever; acute renal failure; anaphylaxis; and perforated diverticulitis.

	Overall (n = 254)
Sepsis	24% (62)
Arrhythmia	12% (30)
Tachycardia (not sinus or AFib)^ a^	6% (15)
AFib with RVR^ b^	2% (5)
Bradycardia	2% (5)
Sinus tachycardia	1% (3)
Type 1 second-degree AV block^ c^	1% (2)
Hypoxia	10% (26)
Electrolyte abnormality, DKA/HHS^ d^	9% (22)
Non-GI and GI bleed, anemia^ e^	9% (22)
Other^ f^	7% (17)
Concern for acute coronary syndrome	6% (16)
Pre-syncope, syncope	6% (16)
Seizure, altered mental status	4% (11)
Opiates/ alcohol withdrawal	4% (9)
Non-massive Transfusion	3% (7)
Hypertensive urgency/ emergency	2% (5)
Decompensated heart failure	2% (4)
Hypotension	2% (4)
Concern for pulmonary embolus	1% (3)

Telemetry duration

The duration of telemetry ranged from 1hr:4min to 759hr:39min with 14% of patients on CCM for less than 24 hours, and 62% on CCM for more than 48 hours (of which 56% was on CCM for 48-96 hours, 31% for 96-192 hours; and 13% for more than 192 hours). The total duration of CCM for 7% of patients included time in the intensive care unit.

Telemetry results

Of the cumulative total of more than 20,573 hours of CCM provided to this patient population, 37% of patients demonstrated only a normal sinus rhythm and 3% (n = 8) had a clinically significant arrhythmia that affected management. One patient with known atrial fibrillation (AFib) presented with rapid ventricular rate (RVR) and later developed a nine-second pause that was observed on telemetry, prompting pacemaker implantation for tachycardia-bradycardia syndrome during that hospitalization. One patient, with a biventricular implantable cardioverter defibrillator presented with persistent diarrhea in the setting of a prolonged corrected QT interval of 536 msec, then developed an episode of sustained ventricular tachycardia that was observed on CCM. Five patients, none of which were noted to be septic, developed new-onset AFib/ flutter that was detected on telemetry. One patient developed new asymptomatic paroxysmal atrial tachycardia while on CCM for the indication of hypoxia and was subsequently initiated on therapy with metoprolol. Of the eight patients with a clinically significant arrhythmia that affected management, six patients had been initiated on CCM for cardiac-related reasons and the remaining two had been initiated on CCM for hypoxia. Table 3 details the arrhythmias detected in the study population.

**Table 3:** Results of continuous cardiac monitoring The arrhythmias observed on telemetry for the patient population are listed in descending order of frequency. *^a^*Seven of the 11 patients with rate-controlled AFib/flutter had a known history of AFib/flutter. *^b^*Four of the six patients with AFib with RVR had a known history of AFib. *^c^*Arrhythmias in the "Other" category included: accelerated junctional rhythm; bigeminy; trigeminy; nine-second pause; atrial ectopy; and bundle branch block.

	Overall (n = 254)
Normal sinus rhythm only	37% (94)
No comments noted about telemetry results	31% (79)
Sinus tachycardia	11% (28)
Atrial fibrillation (AFib) or flutter	6% (17)
Rate-controlled	4% (11)^ a^
with rapid ventricular rate (RVR)	2% (6)^ b^
Premature atrial or ventricular contractions	6% (15)
Bradycardia	6% (15)
Tachycardia (not sinus or AFib)	5% (12)
Other^ c^	4% (10)
Atrioventricular block	3% (7)
Paced rhythm	3% (7)
Non-sustained ventricular tachycardia	2% (6)
Ventricular tachycardia	0.4% (1)

Telemetry discontinuation

Thirty-nine percent of the CCM orders were not discontinued until hospital discharge.

## Discussion

The results of this study provide insight into how telemetry is utilized by hospitalist-led IM resident-staffed inpatient teaching teams at a single institution and identifies potential areas for quality improvement intervention to decrease telemetry overutilization without negatively impacting patient care. This study is unique in that it examines patients who were initiated on CCM during their hospital course by a hospitalist-led inpatient teaching team, and not on admission.

Twenty-four percent of the patients in our study (n = 62) were initiated on telemetry for the indication of sepsis, likely to monitor for the development of new-onset AFib among other reasons. New onset AFib in the setting of sepsis is associated with adverse outcomes, including a higher rate of AFib reoccurrence after hospital discharge, ICU mortality, ICU and in-hospital length of stay, and ischemic stroke [10-11]. Among these 62 patients, no clinically significant arrhythmias, including AFib, were detected over a cumulative total of 203 days of CCM. Sepsis affects more than 659,000 Americans annually, so it would be cost-effective to be able to identify those patients who are most likely to develop new-onset AFib and determine the duration that they need to be monitored on CCM [12]. Gandhi et al. found that in sepsis, new-onset AFib occurred more in the elderly, those with a prior history of a cardiovascular and respiratory disease, and those with increased severity of illness [11]. Similarly, Kupiers et al. found that white race, organ failure, and pulmonary artery catheter use were independent risk factors for new-onset AFib in sepsis [13].

The finding that 10% of the patients (n = 26) were initiated on telemetry for the indication of hypoxia identifies an area for potential intervention to decrease telemetry over-utilization in the inpatient setting. At our institution, continuous pulse oximetry (CPOx) monitoring was only possible in a telemetry-capable bed at the time of the study. Thus, it may be economical to invest in remote CPOx monitoring so that the telemetry-capable beds are reserved for patients who require CCM, and not CPOx exclusively. Of note, however, much of the literature on CPOx usage has been among ICU and surgical patients so limited data exists on the indications for CPOx monitoring among non-ICU general medical patients. Like CCM, CPOx is also a limited resource that has the potential to be overutilized.

Data on which non-ICU general medical patients could potentially benefit from CCM is limited. In a review of the literature, Chen and Hollander found evidence for CCM in patients with an acute cerebrovascular event or massive blood transfusion (defined as at least 10 units of packed red blood cells within 24 hours) [14]. They identified a potential benefit for CCM in patients with syncope and multiple risk factors for an arrhythmic cause; in cirrhotic patients with variceal hemorrhage that received vasopressin or neuroleptics for procedural sedation; and in patients with abnormal serum potassium levels (hyperkalemia with attributable ECG changes; potassium > 6.5 mmol/L; hypokalemia with digoxin toxicity) or prolonged QT interval [14]. There was no evidence to support CCM in stable patients with acute or chronic anemia requiring a non-massive blood transfusion [14]. The AHA recently published an update to their recommendations on indications for ECG monitoring. Among general medicine patients, continuous ECG monitoring was recommended for patients with acute ischemic stroke (for up to 24 to 48 hours after admission or longer in the setting of cryptogenic stroke); moderate to severe potassium or magnesium abnormalities; and overdose with psychotropic drugs, methadone, inhalants, or cocaine [15].

While the results of this study add a unique perspective to our understanding of telemetry utilization among hospitalist-led inpatient teaching teams, there were several limitations in the study design and implementation. The study is a retrospective chart review and relied entirely on available documentation and order entries for data collection. Further, this is a single academic institution study. Thus, the results are not representative of and cannot be generalized to all populations. Further, no data were obtained on patients who were initially admitted to one of the general IM teaching teams under a LOC of "telemetry." This would have further enriched the sample population and would have made it possible to determine if there were any differences in management and/or outcomes between those who were initially admitted to a telemetry-capable bed and those who were subsequently initiated on CCM later in their hospital stay.

Future studies could use these study results as pre-intervention data for a quality improvement intervention aimed at decreasing the overutilization of telemetry at our institution. The intervention could potentially include daily “telemetry rounds” where hospitalists and their resident-staffed teams review the decision to continue or discontinue CCM for their patients; formal education of hospitalists and residents on the existing evidence of benefit, or lack thereof, for CCM among specific general medical patients; and/or an automatic expiration of orders for CCM after 48 hours in the electronic medical records unless a physician renews the existing order prior to expiration and updates the indication for CCM. Alternatively, future studies could further examine the factors that influence hospitalist-led inpatient teaching teams to initiate CCM for patients who have sepsis, which was the reason for 24% of the patients in this study.

## Conclusions

This study provides valuable information on practice patterns associated with CCM among hospitalist-led inpatient teaching teams in a large, urban, academic medical center. Findings highlight the wide spectrum of reasons for telemetry initiation; the not uncommon practice of maintaining patients on CCM for more than 48 hours; and the low yield in terms of clinically significant arrhythmias detected in this patient population.
